# 29 Juvenile idiopathic arthritis and osteogenesis imperfecta: an exceptional association

**DOI:** 10.1093/rheumatology/keac496.025

**Published:** 2022-10-07

**Authors:** Hannech Emna, Boussaid Soumaya, Rekik Sonia, Jemmali Samia, Rahmouni Safa, Ajlani Houda, Sehli Hela, Elleuch Mohamed

**Affiliations:** 1 Rheumatology Department, La Rabta Hospital, Tunis, Tunisia; 2 Faculty of Medicine of Tunis, University Tunis El Manar

## Abstract

**Background:**

Juvenile idiopathic arthritis (JIA) is the most common chronic rheumatic disease of unknown aetiology in childhood and predominantly presents with peripheral arthritis. Osteogenesis imperfecta (OI) is an autosomal dominant inherited disease defined by bone fragility due to abnormal collagen synthesis. It affects the entire skeleton, predisposing the patient to fractures.

We report an uncommon association between osteogenesis imperfecta and juvenile idiopathic arthritis.

**Case presentation:**

A 15-year-old child presented to our rheumatology department with medical history of recurrent bone fractures due to low-energy trauma since the age of five years. The patient was born from a consanguineous marriage. He has been diagnosed with a polyarticular JIA since the age of eight years-old. The initial clinical presentation was a symmetric, cumulative, large and small joint polyarthritis. The rheumatoid factor was negative. No other autoantibodies were detected.

The patient was treated initially with methotrexate which was inefficient. He developed joint deformities. Then, biologic therapy was associated with a good response.

Clinical examination on entry showed severe joint deformities touching elbows, wrists and interphalangeal joints. Arms and thighs were curved and an unequal leg length was noted. There was no arthritis. The patient has bluish sclera. Neurological and dental examination was normal. There was no hearing loss. Laboratory findings showed a normal C-reactive protein. Calcium and phosphate serum levels were within normal ranges. On radiological investigations, radiographs showed excessive trabecular bone transparency, cortical bone thinning and incomplete bone fractures. Bone densitometry revealed a Z-score of the lumbar spine of -4.2 SD.

According to clinical, biological, and radiological investigations, the diagnosis of JIA associated with OI was confirmed clinically. Treatment with Pamidronate was initiated intravenously.

**Conclusion:**

We reported an exceptional association between JIA and OI which occurred in a young Tunisian patient. We emphasize that this association should be considered when severe joints and extremities deformities occur.

Funding: No specific funding was received from any bodies in the public, commercial or not-for-profit sectors to carry out the work described in this abstract.

Acknowledgements: declared none.

